# Continuation of injectable contraception when self-injected vs. administered by a facility-based health worker: a nonrandomized, prospective cohort study in Uganda^☆^^☆☆^

**DOI:** 10.1016/j.contraception.2018.03.032

**Published:** 2018-11

**Authors:** Jane Cover, Allen Namagembe, Justine Tumusiime, Damalie Nsangi, Jeanette Lim, Dinah Nakiganda-Busiku

**Affiliations:** a PATH, PO Box 900922, Seattle, WA 98109, USA; b PATH, PO Box 7404, Kampala, Uganda; c Uganda Ministry of Health, PO Box 7272, Kampala, Uganda

**Keywords:** Self-administration, Self-injection, Discontinuation, DMPA-SC, Sayana® Press

## Abstract

**Objective:**

The purpose of this study was to compare 12-month continuation rates for subcutaneous depot medroxyprogesterone acetate (DMPA-SC) administered via self-injection and DMPA-IM administered by a health worker in Uganda.

**Study design:**

Women seeking injectable contraception at participating health facilities were offered the choice of self-injecting DMPA-SC or receiving an injection of DMPA-IM from a health worker. Those opting for self-injection were trained one-on-one. They self-injected under supervision and took home three units, a client instruction guide and a reinjection calendar. Those opting for DMPA-IM received an injection and an appointment card for the next facility visit in 3 months. We interviewed participants at baseline (first injection) and after 3 (second injection), 6 (third injection) and 9 (fourth injection) months, or upon discontinuation. We used Kaplan–Meier methods to estimate continuation probabilities, with a log-rank test to compare differences between groups. A multivariate Cox regression identified factors correlated with discontinuation.

**Results:**

The 12-month continuation rate for the 561 women self-injecting DMPA-SC was .81 [95% confidence interval (CI) .78–.84], and for 600 women receiving DMPA-IM from a health worker, it was .65 (95% CI .61–.69), a significant difference at the .05 level. There were no differences in pregnancy rates or side effects. The multivariate analysis revealed that, controlling for covariates, self-injecting reduced the hazard for discontinuing by 46%. A significant interaction between injection group and age suggests that self-injection may help younger women continue injectable use.

**Conclusions:**

The significant difference in 12-month continuation between women self-injecting DMPA-SC and women receiving DMPA-IM from a health worker — which remains significant in a multivariate analysis — suggests that self-injection may improve injectable contraceptive continuation.

**Implications:**

While injectable contraceptives are popular throughout much of sub-Saharan Africa, they have high rates of discontinuation. This study is the second from an African country to demonstrate that self-injection may improve injectable continuation rates and may do so without increasing the risk of pregnancy or adverse events.

## 1 Introduction

Injectable contraceptives are a popular method for preventing pregnancy, especially in sub-Saharan Africa [1]. However, the injectable is notable among contraceptive methods for high rates of discontinuation. While side effects are the most common reason given for discontinuation, another possible cause is the challenge of the reinjection schedule because women using depot medroxyprogesterone acetate (DMPA) must return to the clinic or a community health worker every 3 months for injections [2]. Discontinuation is a significant cause of unmet need, with a recent analysis of Demographic and Health Surveys data from 36 countries estimating the proportion of unintended births attributable to discontinuation at one-third of the total in all the countries [3]. Thus, methods or delivery modalities that facilitate continuation are an essential part of addressing unmet need.

A new formulation and presentation of DMPA administered subcutaneously (DMPA-SC) is now approved and available in at least 25 countries worldwide (Sayana® Press, Pfizer Inc.). This product, which is packaged in an all-in-one injection system and designed to be easy to use, can be given by low-level providers or women themselves through self-injection, and it is highly acceptable relative to the intramuscular version (DMPA-IM) [4], [5]. By reducing access challenges, self-injection of DMPA-SC may facilitate continuing use of injectable contraception, particularly for women who live far from health facilities.

In 2015, the UK Medicines and Healthcare products Regulatory Agency approved Sayana® Press for self-injection, and in 2016, the National Drug Authority in Uganda followed suit. Previous research from Uganda, Senegal and other countries found self-injection to be feasible and acceptable, but data on whether self-injection improves contraceptive continuation are more limited—especially in low-resource settings [6], [7]. A study from Scotland found no difference in 12-month continuation rates between women self-injecting DMPA-SC and women receiving DMPA-IM injections from a health worker [8], while a randomized clinical trial in the United States found no difference in continuation when both groups were using DMPA-SC, either self- or provider-administered [9]. Based on this evidence, a systematic review of the literature concluded that “there may be little or no difference in continuation” [10]. These studies raise questions as to whether offering self-injection as a delivery modality would enable women in low-resource settings to use injectable contraception more continuously.

The current study intended to compare 12-month continuation rates for women self-injecting with DMPA-SC and women receiving DMPA-IM from a health worker. The secondary objectives were to identify differences in characteristics between women who chose self-injection and those who chose injection from a health worker and to identify factors that contributed to discontinuation for injectable users.

## 2 Materials and methods

### 2.1 Study design, sites and participants

This was a nonrandomized cohort study conducted at 14 public-sector health facilities in five districts in Uganda. Nine of the facilities were Health Center II, serving parish-level populations of up to 5000 people with essential health services. Two facilities were Health Center III (each serving about 20,000 people), and three were hospitals [11]. Data were collected from April 27, 2016, through July 24, 2017.

Women who participated were 18 to 45 years of age and eligible to receive injectable contraception per World Health Organization (WHO) medical eligibility guidelines. Anyone who did not reside permanently in the area, felt unwell on the day of enrollment, did not wish to avoid pregnancy for a minimum of 12 months, did not speak the primary language of the area or was not able to provide informed consent was excluded from the study.

### 2.2 Study procedures

#### 2.2.1 Enrollment visit

Licensed nurses implemented the study. They were trained in DMPA-SC and DMPA-IM administration, research ethics and informed consent, interviewing techniques and how to train women to self-inject. All women attending participating health facilities for routine family planning visits who expressed an intention to use injectable contraception (whether new, continuing or past injectable users) were first assessed for study eligibility. Eligible participants provided informed consent, and we conducted urine pregnancy tests to confirm that these participants were not pregnant. Two groups were then enrolled: those who opted to try self-injection of DMPA-SC and those who chose DMPA-IM administered by a health worker. Those who opted for self-injection were trained one-on-one and administered their first injection under the supervision of a study nurse. They were instructed to reinject every 3 months and advised that they could reinject up to 14 days early and 29 days late, as per the WHO-approved reinjection window for DMPA [12]. They were given an instruction booklet, reinjection calendar and three units to take home. Women who opted for DMPA-IM received their first injection from a study nurse, were instructed to return in 3 months and were given an appointment card.

#### 2.2.2 Follow-up visits

We interviewed participants following each scheduled injection date for up to 9 months (three reinjections or the equivalent of 12 months of contraceptive coverage). Women in the DMPA-IM group were interviewed in a private setting at each return visit to the health facility after receiving the injection from a health worker. If women in the DMPA-IM group did not return for reinjection within 29 days of their reinjection date, we followed up with an interview at home or other convenient location. Similarly, those in the self-injection group were followed up at home 29 days after each scheduled reinjection date. We asked about whether they were continuing injections and their reinjection date; their experience of side effects, self-injection or a return clinic visit; satisfaction with the method; and intention to continue. Women who discontinued were asked their reasons for discontinuation.

### 2.3 Sample size

For the primary objective, the target sample size was 604 in each group, assuming a power of 90%, a significance level of .05 (two-sided test) and a 10-percentage-point difference in continuation rates between DMPA-IM users and those self-injecting DMPA-SC. The sample size anticipated a loss to follow-up rate of 20%.

### 2.4 Data collection and analysis

Nurses conducted private, face-to-face interviews in English or the primary language spoken in the area and entered responses electronically via cell phones. A team leader monitored data collection in each district and downloaded data on a biweekly basis to assess quality and completeness.

Data analysis was performed using Stata, version 14. We considered women lost to follow-up, women who changed their minds about self-injection at any time after consenting and women judged not competent with the injection technique^1^ to have discontinued the study. Consistent with an intent-to-treat approach, women who switched from DMPA-IM to DMPA-SC injected by a health worker were considered to have continued (in the DMPA-IM group) since they had switched from one type of provider-administered injectable to another.

While study staff were instructed to discontinue women if they gave or received an injection after the reinjection window had closed, 21 subjects were allowed to continue in the study despite being late. During analysis, these cases were reclassified as discontinued based on the dates of reinjection.

We tabulated estimates of cumulative contraceptive continuation over the 12-month period using Kaplan–Meier methods, with continuation censored for those who received their injection at 9 months (12 months of contraceptive coverage). The log-rank test was used to estimate equality of the survival function between injection groups. Differences between groups in background characteristics and side effects were evaluated with *χ*^2^ tests or *t* tests, employing a significance threshold of .05 for a two-sided test. Cox proportional hazard models were used to identify significant predictors of discontinuation.

### 2.5 Safety monitoring

We advised women to contact the study nurse or the health worker if they experienced any adverse events. The study nurse alerted the district medical monitor, a licensed gynecologist engaged by the study, for any suspected serious adverse event. The medical monitor was instructed to follow up with the participant within 24 h and report to the principal investigator within 24 h of the examination.

### 2.6 Confidentiality and ethical approvals

PATH's Research Ethics Committee, Mulago Hospital Institutional Review Board and the Uganda National Council for Science and Technology granted approval to conduct this research study.

## 3 Results

**Fig. 1:**
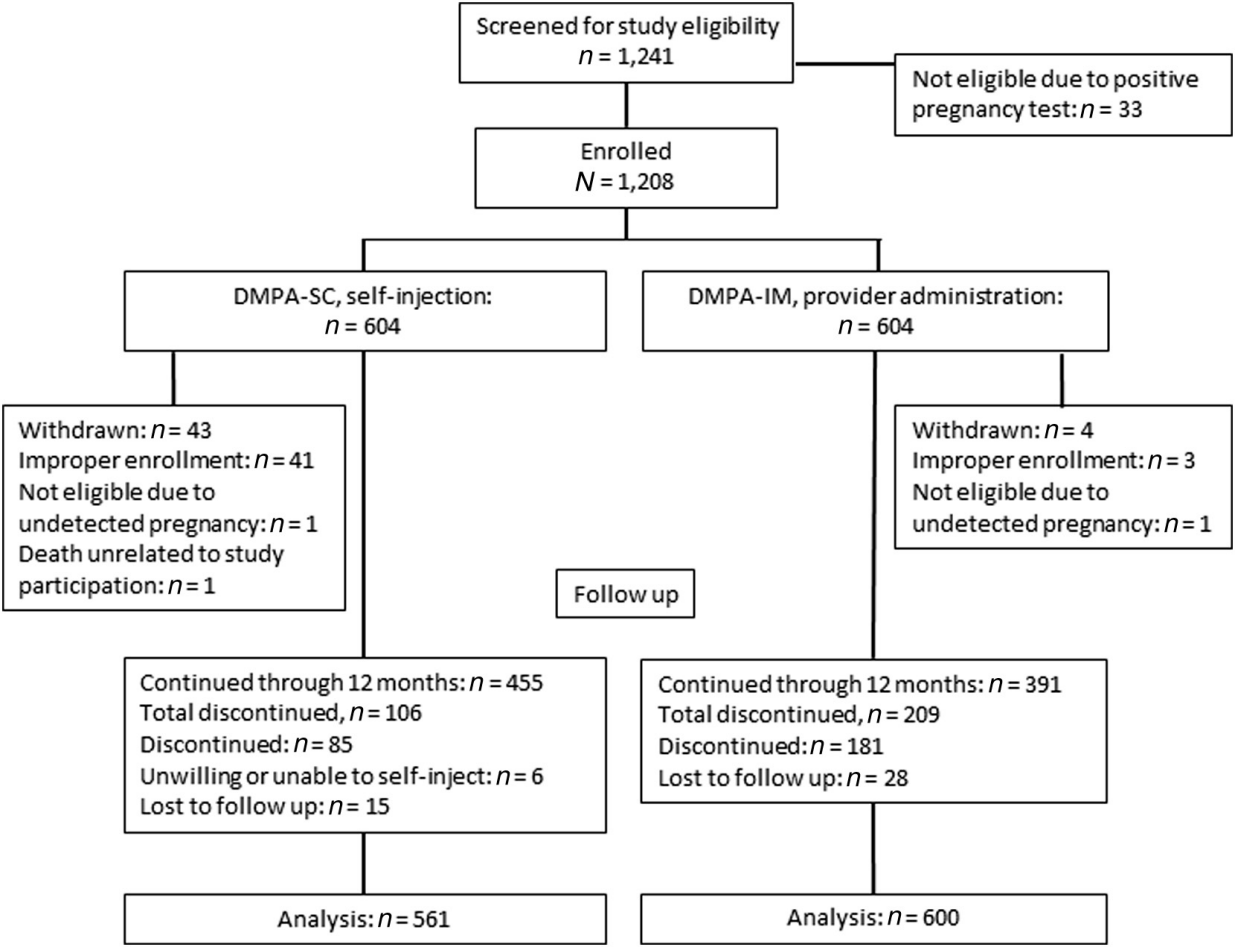
Participant flow diagram.

In all, 1161 women participated: 561 in the DMPA-SC self-injection group and 600 in the provider-administered DMPA-IM group (Fig. 1). The target sample size was not reached because a study site was closed during enrollment (and participants withdrawn) due to lack of adherence to training and enrollment procedures. In addition, two participants were enrolled but later withdrawn due to undetected pregnancy (screening failures), with fetal age determined by ultrasound. One serious adverse event occurred in a participant in the DMPA-SC group (death), which was determined by the PATH and Mulago IRBs to be unrelated to study participation. Forty-three women were lost to follow-up and considered to have discontinued 90 days after their last recorded injection date. Thirty-three women in the DMPA-IM group switched to DMPA-SC administered by a health worker and were retained in the DMPA-IM group as continuers. Six women declined to self-inject after participating in training, and these were considered to have discontinued on day 1.

### 3.1 Participant background

Relative to those who chose provider injections, women who chose self-injection were significantly more likely to have a secondary or higher level of education, be engaged in salaried employment and have more household assets (Table 1). More self-injectors made household and family planning decisions jointly with a partner and came from communities and families that support family planning use. More self-injectors had used DMPA-SC administered by a health worker in the past and had visited a community health worker for contraception. In addition, fewer self-injectors reported needle anxiety.

**Table 1:** Participant characteristics

	Self-injected DMPA-SC (*n*=561)		Provider-injected DMPA-IM (*n*=600)		Sig.
	% or mean	*n*	% or mean	*n*	p
Mean age (SD)	26.9 (6.4)	561	26.5 (6.2)	600	.31
Married or cohabiting	82.5	463	80.8	485	.46
Mean parity (SD)	3.1 (2.0)	561	3.1 (1.8)	600	.17
Education level					
None	7.5	42	9.7	58	
Primary	70.2	394	73.3	440	
Secondary or more	22.3	125	17.0	102	.04
Working outside the home	75.8	425	76.5	459	.80
Collects paycheck	11.1	47	7.2	33	.05
Mean number of household assets (SD)	6.1 (2.7)	561	5.7 (2.4)	600	.00
Mean travel time RT to facility (SD)	107.3 (86.9)	555	116.0 (91.1)	600	.10
Paid to travel to facility	13.7	77	15.0	90	.59
First-time contraceptive user	14.1	79	15.5	93	.49
Current or past injectable user	79.7	447	77.8	467	.44
Current or past DMPA-SC user	36.7	206	8.2	49	.00
Injection anxiety					
Low	89.1	500	87.5	525	
Moderate	10.3	58	8.0	48	
High	0.5	3	4.5	27	.00
Mean number of methods used (SD)	1.6 (1.2)	561	1.5 (1.1)	600	.08
Previously visited CHW for contraception	22.5	126	17.0	102	.02
Community supports contraceptive use					
Almost all	25.0	140	19.0	114	.02
Most	39.2	220	45.2	271	
Some	24.8	139	25.3	152	
Very few/none	11.1	62	10.5	63	
Friends, family support contraceptive use					
Almost all	28.3	159	23.0	138	.04
Most	37.6	211	40.3	242	
Some	21.6	121	24.2	145	
Very few/none	12.5	70	12.5	75	
Husband supports use of family planning	81.6	458	76.3	458	.03
Family planning decisions made jointly	66.3	372	59.0	354	.01
Mean joint decision-making scale (SD)	2.2 (1.8)	561	2.0 (1.7)	600	.01

Abbreviations: CHW, community health worker; RT, roundtrip; SD, standard deviation.

### 3.2 Continuation

**Fig. 2:**
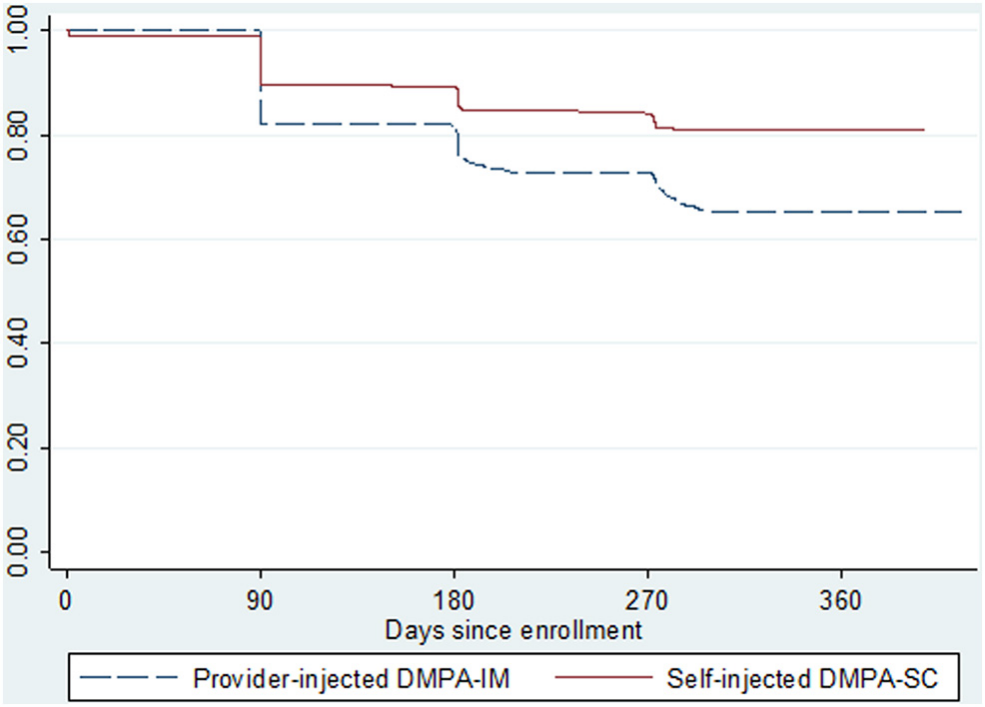
Kaplan–Meier cumulative probability of continuation. Log-rank test for equality of survivor function, p value=.0000.

The probability of continuation was significantly higher for women in the self-injection group compared with women in the DMPA-IM group at each time point after the first injection (Fig. 2). By 12 months, the cumulative probability of continuing was .81 in the self-injection group [confidence interval (CI)=.78–.84] and .65 in the provider-administered group (CI=.61–.69). A sensitivity analysis (results not shown) in which those lost to follow-up were excluded from analysis also found significantly greater probability of continuation in the self-injection group.

Three women in the self-injection group and two in the DMPA-IM group became pregnant during the study; this difference was not significant.

### 3.3 Determinants of discontinuation

Turning to the factors associated with discontinuation (Table 2), the main effects multivariate analysis (first panel) revealed a greater risk of discontinuation for women recruited from facilities in more rural locations, the districts of Apac and Oyam, compared with Gulu (not shown), and greater risk for women age 18 to 24 years. Factors that reduced the risk of discontinuation included having a primary or secondary education, having a partner who supported use of family planning and having more children.

**Table 2:** Cox proportional hazard ratios with clustering by study site, predicting risk of discontinuation over 12 months

	Main effects model		Interaction model	
	Hazard ratio	p	Hazard ratio	p
Injection group (self-injected=1)	0.54 (0.44–0.68)	.00	0.75 (0.56–0.99)	.05
Clinic in a rural area	1.71 (1.23–2.39)	.00	1.71 (1.21–2.43)	.00
Education (reference, none)				
Primary	0.54 (0.42–0.68)	.00	0.53 (0.41–0.69)	.00
Secondary or greater	0.41 (0.23–2.39)	.00	0.41 (0.29–0.58)	.00
Parity (number of children)	0.89 (.81–0.99)	.03	0.88 (0.80–0.98)	.02
Husband supports family planning use	0.70 (0.55–0.89)	.00	0.70 (0.55–0.89)	.00
Youth age 18–24 years	1.25 (1.01–1.54)	.04	1.51 (1.16–1.97)	.00
Group * youth			0.53 (0.35–0.79)	.00

Multivariate analyses also include measures of the districts where sites were located (not shown).

Likelihood-ratio test for interaction over main effects model: LR *χ*^2^(1) = 7.03 Prob > *χ*^2^=0.0080.

Despite significant differences between women who opted for self-injection and women who selected DMPA-IM (recall Table 1), the adjusted hazard ratio indicated that self-injection was associated with a significantly reduced risk of discontinuation, controlling for a host of confounding variables. Self-injecting reduced the hazard by 46%.

As shown in the second panel of Table 2 (interaction model), the significant interaction effect indicated that the effect of injection group varied by age (log-likelihood *χ*^2^ test=7.03, p<.01). For those 25 and older, self-injecting reduced the risk of discontinuation by 25%, while for youth ages 18 to 24 years, self-injecting reduced the risk by 40%. Youth in the DMPA-IM group had a 50% greater hazard of discontinuing relative to older women.

### 3.4 Reasons for discontinuation

Among women who discontinued, the reasons offered differed somewhat between the two groups (Table 3). The most common explanation for discontinuing in the self-injection group — offered by about 25% — was husband disapproval, followed by challenges with self-injection (23%) and forgetting or being late to reinject (22%). The most common reason offered in the DMPA-IM group was forgetting or being late for the reinjection visit (37%), followed by difficulty returning to the clinic or obtaining the method upon return to the facility (26%).

**Table 3:** Reasons for discontinuing

	Self-injected DMPA-SC (*n*=91)		Provider-administered DMPA-IM (*n*=181)	
	%	*n*	%	*n*
Husband disapproval	25.3	23	9.4	17
Challenges with self-injection	23.1	21	–	–
To have a child	12.1	11	7.2	13
Forgot/late for injection	22.0	20	37.0	67
Access challenges/stockouts	3.3	3	26.0	47
No sexual relations	3.3	3	18.8	34
Side effects	7.7	7	16.0	29
Got pregnant	3.3	3	1.1	2
Developed contraindications	3.3	3	1.7	3
Distrust the method/rumors	0.0	0	3.3	6

### 3.5 Experience of side effects

While the percentage of women reporting side effects was greater among DMPA-IM users at each point in time, the difference was not significant (Table 4). With respect to injection site reactions (ISRs), women in the DMPA-SC group consistently reported significantly more ISRs than women receiving DMPA-IM. The most common ISR reported among self-injectors was a dimple or indentation while DMPA-IM users reported itching (data not shown). The ISRs were not severe, and very few women in either group sought advice or treatment.

**Table 4:** Experience of side effects and ISRs

	After 1st injection		After 2nd injection		After 3rd injection	
	Self-injected (*n*=539)	DMPA-IM (*n*=580)	Self-injected (*n*=497)	DMPA-IM (*n*=489)	Self-injected (*n*=473)	DMPA-IM (*n*=432)
Reported side effects	161 (29.9)	197 (34.0)	117 (23.5)	135 (27.6)	88 (18.6)	98 (22.7)
Sought advice for side effects	48 (8.9)	57 (9.8)	33 (6.6)	47 (9.6)	35 (7.4)	36 (8.3)
Reported ISR	33⁎ (6.1)	8 (1.4)	25⁎ (5.0)	8 (1.6)	38⁎(8.0)	5 (1.2)
Sought advice for ISR	0 (0.0)	2 (0.3)	2 (0.4)	0 (0.0)	3 (0.6)	2 (0.5)

⁎ Significant at the p<.05 level.

## 4 Discussion

The results of this study of women using self-injection in a low-resource setting align with recent studies from Malawi and the United States that found similarly elevated 1-year continuation rates for self-injectors relative to those receiving DMPA injections from health workers [13], [14]. Collectively, these studies, which have comparable rates of unintended pregnancy between groups and lack serious adverse events related to study participation, add to the now considerable evidence that women from many walks of life can self-inject safely [6], [7], [15], [16]. The findings suggest that self-injection may improve outcomes for younger women, who are subject to high rates of discontinuation [17], [18], [19].

The findings also suggest that early adopters of self-injection may be more educated, be more empowered and have more familial support than those who choose DMPA-IM from health workers. Appreciating the contribution of a supportive husband or partner for longer injectable use will be important for the family planning program in Uganda.

The prominence of forgetting or lateness as a reason for discontinuation for DMPA-IM users may be addressed by providing complete information about the WHO reinjection window, which permits reinjection up to 4 weeks late. It is not the customary practice in Uganda to inform women of the full window, possibly due to fears that women will postpone their return visit. This is counterproductive if women who miss their appointment date discontinue rather than travel to the facility after the date has passed. For self-injectors, mechanisms to support women who are self-injecting at home may reduce discontinuation caused by challenges with injection technique and forgotten or mistimed reinjection.

That women who self-inject DMPA-SC may experience more ISRs than DMPA-IM users has implications for program planners. The predominant type of reaction among DMPA-SC users (dimple) and the nonseverity of ISRs suggest that the difference may be due to the type of injection (subcutaneous) rather than the practice of self-injection. Indeed, the original clinical trials for DMPA-SC also found higher frequency of ISRs among those receiving DMPA-SC from a provider compared with those administered DMPA-IM [20]. Nonetheless, as more programs integrate DMPA-SC into their method mix, planners will need to address concerns over ISRs, lest self-injectors wrongly attribute the ISR to faulty technique and discontinue use.

### 4.1 Limitations of the study

Women who participated in this study self-selected into either the self-injection DMPA-SC group or the provider-administered DMPA-IM group. Because participants were not randomly assigned and the multivariate analysis can only control for a limited number of factors, there may be unobserved heterogeneity between groups that could underlie the difference in continuation.

The study was not designed to evaluate systematically the nature or duration of side effects experienced. Anecdotally, women and health workers in Uganda reported fewer side effects with DMPA-SC. Additional research is needed to quantify any difference in frequency or type of side effects.

By virtue of the study recruitment strategy at facilities, this research does not reveal the potential appeal of self-injection for new family planning clients. Whether self-injection will advance the Family Planning 2020 goal of recruiting 120 million new users [21] will become more apparent as countries roll out self-injection programs accompanied by wide-scale demand-generation activities.

This study was designed to compare 12-month continuation rates for two injectable contraceptives with different delivery modalities. It does not address the question of what happens after 12 months, including how well family planning programs ultimately transition discontinuing clients to contraceptive methods that better suit their needs. These important questions merit more attention.

## Funding

This work was supported by the Bill & Melinda Gates Foundation, Seattle, WA, USA, and the Children's Investment Fund Foundation, London, UK. The funders did not play a role in data collection, analysis, interpretation of data, the writing of the report or the decision to submit the article for publication.

## Footnotes

1 Women were evaluated in their injection technique at the time of their first self-injection and, if not competent, were asked to return to the facility for additional training when due for their second injection. If still not competent after additional training, they were discontinued (*n*=2).
